# GOLDEN 2-LIKE Transcription Factors of Plants

**DOI:** 10.3389/fpls.2016.01509

**Published:** 2016-10-04

**Authors:** Min Chen, Meiling Ji, Binbin Wen, Li Liu, Shaoxuan Li, Xiude Chen, Dongsheng Gao, Ling Li

**Affiliations:** ^1^State Key Laboratory of Crop Biology, Shandong Agricultural UniversityTaian, China; ^2^College of Horticulture Science and Engineering, Shandong Agricultural UniversityTaian, China; ^3^Shandong Collaborative Innovation Center for Fruit and Vegetable Production with High Quality and EfficiencyTaian, China

**Keywords:** GLKs, chloroplast development, pathogen resistance, senescence, transcription factors

## Abstract

*Golden2-like* (*GLK*) transcription factors are members of the GARP family of Myb transcription factors with an established relationship to chloroplast development in the plant kingdom. In the last century, *Golden2* was proposed as a second golden producing factor and identified as controlling cellular differentiation in maize leaves. Then, *GLK*s were also found to play roles in disease defense and their function is conserved in regulating chloroplast development. Recently, research on GLKs has rapidly increased and shown that *GLK*s control chloroplast development in green and non-green tissues. Moreover, links between phytohormones and *GLK*s were verified. In this mini-review, we summarize the history, conservation, function, potential targets and degradation of *GLK*s.

## Introduction

In plants, the development of functional chloroplasts is dependent on tight co-ordination between chloroplast and nuclear genomes. Previous studies estimated that almost 3000 nuclear-encoded proteins are located in the chloroplast (Pedro et al., 2012). In the nuclear genome, *Golden2-like* (*GLK*) transcription factors have been shown to be involved in the related expression of nuclear chloroplast-localized proteins and photosynthesis-related genes in maize (*Zea mays*), the moss *Physcomitrella patens* and *Arabidopsis thaliana* (Hall et al., 1998; Rossini et al., 2001; Fitter et al., 2002; Yasumura et al., 2005; Waters et al., 2009). The name, *Golden2*, follows nomenclature used for the first golden producing factor, *golden1*, found in maize by Jenkins (1926).

## History

Golden2 was named in maize almost 90 years ago and subsequently researchers found many *Golden2-like* (*GLK*) genes in *Arabidopsis*, rice (*Oryza sativa*), moss (*P. patens*), pepper (*Capsicum annuum*) and tomato (*Solanum lycopersicum*; Fitter et al., 2002; Bravo-Garcia et al., 2009; Powell et al., 2012; Brand et al., 2014). Then, *Golden2*, with TEA DNA-binding domains, was shown to encode a 2.2-kb transcript mainly in maize leaves. In addition, *Golden2* plays a critical role in cellular differentiation (Hall et al., 1998); and *GLK* expression in maize, rice and *Arabidopsis* is regulated by light (Rossini et al., 2001; Fitter et al., 2002), while *GLK*s in *Brassica* spp. are induced by cold stress (Savitch et al., 2005). Subsequently, *Golden2* was classified into the GARP family, named by Riechmann et al. (2000). In most characterized plant genomes, including *Arabidopsis*, maize, rice, sorghum (*Sorghum bicolor*), and *P. patens* (i.e., from bryophytes to angiosperms), *GLK* genes exist as pairs.

## Evolution and Conservation of *GLK*s in Diverse Plants

Land plants have 1–4 *GLK*s but no *GLK*s have been found in sequenced algal genomes (Wang et al., 2013). Wang et al. (2013) also demonstrated that the ancestral state of flowering plants was a single *GLK* gene and that gene duplication occurred in specific species. *P. patens* contains two *GLK*s due to a recent genome duplication within this species (Yasumura et al., 2005; Rensing et al., 2008). In the C_4_ plants, maize, and sorghum, *GLK1* and *GLK2* are expressed in bundle sheath (BS) and mesophyll (M) cells, respectively, associated with chloroplast dimorphism. However, in *Cleome gynandra*, another C_4_ plant, *CgGLK1* and *CgGLK2* are both expressed in BS and M cells and expression is higher in M cells. Of the identified C_4_ plants, compartmentalization of *GLK* function is not necessary in the development of chloroplast (Wang et al., 2013). Although the function of *GLK*s is conserved, there has been specialization of the *GLK* pathway (Bravo-Garcia et al., 2009). During the evolution from *P. patens* to *Arabidopsis*, the upstream and downstream targets of *GLK*s may have diverged.

*GLK* genes contain two highly conserved domains: a C terminal GCT-box which is specific to *GLK* genes and a DNA-binding domain (DBD) at the C terminal (Rossini et al., 2001). The AREAEAA hexapeptide sequence at the DBD is highly conserved among the GARP family (Hosoda et al., 2002). This DBD occurs in green algae and land plants, whereas GCT-box is found only in land plants.

## Function

### Cellular Differentiation

In all C_4_ plants, there is differentiation of three photosynthetic cell-types in leaf blades: C_4_ BS and C_4_ and C_3_ M cells. In maize, *G2* and *ZmGLK1* transcripts accumulate primarily in C_4_ tissues. In the C_3_ model plant rice and *Arabidopsis*, the *GLK*s act redundantly in promoting photosynthetic development (Rossini et al., 2001; Fitter et al., 2002). Therefore, these studies provide support for the idea that *GLK*s not only control cell-type differentiation processes but also play crucial roles in chloroplast development (Riechmann et al., 2000; Rossini et al., 2001).

### Chloroplast Development

*GLK*s play pivotal roles in regulating chloroplast development in diverse plant species. Although the function of *GLK*s is conserved, different genetic mechanisms may operate upstream and downstream of *GLK* function in diverse species. Chloroplast development is an essential process in all plant cells and three types of chloroplasts exist in C_4_ plants. In one of the earliest studies, the size of chloroplasts and the numbers of thylakoid lamellae in *g2* mutants were both smaller than in wild type (Langdale and Kidner, 1994).

*GLK*s are potent positive regulators of chloroplast development in the plant kingdom. It has been verified that *GLK1* is expressed mainly in leaves, while *GLK2* is predominant in fruit. In *Arabidopsis, AtGLK1 AtGLK2* mutants have pale-green photosynthetic tissues, lower levels of *LHCB6* (a light harvesting chlorophyll a/b binding protein) transcripts and an earlier flowering phenotype. There is a similar pale silique phenotype for *AtGLK2* and *AtGLK1 AtGLK2* mutants (Fitter et al., 2002). Overexpression of either *AtGLK1* or *AtGLK2* in the double mutants led to completely restored levels of leaf chlorophyll (Chl) and *LHCB6* transcripts and the time to flowering in a cell-autonomous manner (Waters et al., 2008). Leister and Kleine (2016) identified *genomes uncoupled* (*gun*) mutants as GLK overexpressors. In addition, galactolipid-synthesis genes which affect biogenesis of thylakoid membranes are also regulated by *GLK*s during leaf development (Kobayashi et al., 2014). In tomato, both *SlGLK1* and *SlGLK2* are expressed in leaves, whereas only *SlGLK2* is expressed in fruit and is predominantly expressed in the green shoulder of fruit of the *U* phenotype. This green shoulder is lost in the uniform ripening (*u*) mutant which was bred for evenly ripened fruit. It was demonstrated that *SlGLK2* influences photosynthesis and the chloroplast developmental gradient in immature fruit (Powell et al., 2012; Nguyen et al., 2014). This conclusion was further verified by a promoter expressed later in fruit development. Furthermore, *SlGLK2* expression is partially controlled by light (Powell et al., 2012). Co-suppression of *SlGLK1* only resulted in pale-green leaves but showed no notable differences in fruit, while co-suppression of *SlGLK2* mimicked the *u* mutant (Powell et al., 2012; Nguyen et al., 2014). Overexpression of *SlGLK2* and *SlGLK1* resulted in uniformly darker green unripe fruit and enhanced nutritional quality in ripe fruit (Powell et al., 2012; Cheng and Lai, 2013; Nguyen et al., 2014). However, overexpression of *GLK1* and *GLK2* do not affect overall ripening regulation according to RNA-seq analysis, showing that the *GLK*s have no impact on general ripening control systems. In pepper, the role of *CaGLK2* in regulating fruit development, and *CaGLK2* expression throughout the entire fruit, has been studied (Brand et al., 2014). Furthermore, the *KNOTTED1-LIKE HOMEOBOX* (*KNOX*) genes act upstream of *SlGLK2*, implying that a regulatory mechanism of chloroplast development exists in fruit (Nadakuduti et al., 2014). All these suggest that *GLK1* and *GLK2* have functional equivalence and are tissue-specific. Interestingly, the tissue-specificity of *SlGLK1* was demonstrated relating to the different Histone 3 Lysine 4 trimethylation (H3K4me3) levels in the promoter region of *SlGLK1*, whereas the equivalent *SlGLK2* locus was not detected (Nguyen et al., 2014).

Plant roots are generally non-green and heterotrophic organs. Roots of some epiphytic plants are green and perform active photosynthesis; however, in most cases, the plants still depend on aerial leaves for energy (Aschan and Pfanz, 2003). The auxin signaling pathway was shown to be involved in regulating chloroplast development through *GLK*s in fruit and also in roots (Kobayashi et al., 2012). The expressions of *GLK1* and *GLK2* are much lower in roots than in leaves (Fitter et al., 2002). Overexpression of *GLK*s not only results in derepressing chloroplast development but also triggers ectopic development of chloroplasts in roots. Although *GLK* overexpression (*GLK_OX_*) can enhance chloroplast development and induce chloroplast division, other plastids including amyloplasts were not affected in *GLK1_OX_* roots. Moreover, carbon dioxide fixation and phototrophic performance of *GLK_OX_* roots increased, implying that root photosynthesis may influence the effective carbon utilization in plants (Kobayashi et al., 2013). In *GLK1_OX_* and *GLK2_OX_* roots, *DGD1* was obviously up-regulated together with *CHLH*, which both encode key enzymes of digalactosyldiacylglycerol synthesis. Kobayashi et al. (2014) also reported that genes involved in fatty acid desaturation were upregulated in *GLK1_OX_* roots.

### Biotic Stress

The role of *GLK*s in disease defense is indicated in several studies of *Arabidopsis* (Savitch et al., 2007; Jhadeswar et al., 2014). The *Arabidopsis* genome contains two *GLK*s of the GARP family: *AtGLK1* and *AtGLK2*. *AtGLK1_OX_* in *Arabidopsis* leads to significant up-regulation of genes related to the defense and salicylic acid (SA) signaling pathway, whereas, *PR1* (an indicator of systemic acquired resistance activation) was down-regulated (Savitch et al., 2007). Compared with wild type, *AtGLK1_OX_* plants exhibited stronger resistance to *Fusarium graminearum* and more susceptibility to the virulent oomycete pathogen *Hyaloperonospora arabidopsidis* (*Hpa*) *Noco2* (Jhadeswar et al., 2014). Taken together, these observations confirmed that the *AtGLK1* not only regulates disease defense-related genes, but also plays different roles when various pathogens challenge *AtGLK1_OX_ Arabidopsis*. Following pretreatment with SA, a method used to induce resistance against *Hpa Noco2* (Ryals et al., 1996; Kunkel and Brooks, 2002), *35S:AtGLK1* plants showed only marginally enhanced resistance to *Hpa Noco2*. The *GLK1 GLK2* double-mutant displayed strong resistance to *Hpa Noco2*. This phenomenon was partially influenced by SA accumulation, and not mediated through *NONEXPRESSOR OF PATHOGENESIS-RELATED 1* (Jhadeswar et al., 2014). Pretreatment with SA can marginally reduce resistance in *GLK1 GLK2* plants. In all tested mutants, the SA signaling pathway is functional but does not provide resistance to *Hpa Noco2*. In addition, both the activation of the SA pathway and suppression of the jasmonic acid (JA) pathway are alternatives for resistance to *Hpa Noco2* (Lawton et al., 1995; Clarke et al., 1998; Li et al., 2001; Murray et al., 2002; Rairdan and Delaney, 2002; Argueso et al., 2012; Massoud et al., 2012). Mutants pretreated with JA, the response of JA-treated mutants suggesting that the susceptible *Arabidopsis* to *Hpa Noco2* requires integration of *GLK*s and JA signaling. However, *AtGLK1*-facilitated resistance to *Botrytis cinerea* is independent of JA signaling. In rice, *OsGLK1* may play a role in resistance to pathogen invasion (Nakamura et al., 2009). Recent studies have indicated that AtGLKs also played a positive role in the tolerance to *Cucumber mosaic virus* (Han et al., 2016).

In addition, Nagatoshi et al. (2016) identified that *GLK*s affect ozone tolerance via controlling stomatal movement. The chimeric repressors for *GLK1* and *GLK2* (*GLK1/2-SRDX*) and *GLK1/2* overexpression (*35S:GLK1/2*) were used and the researchers found that *GLK1/2-SRDX* showed remarkable tolerance to ozone with no defects in M chloroplasts, while *35S:GLK1/2* was hypersensitive to ozone. In addition, *GLK*s also affect stomatal aperture and expression of genes controlling movement of stomata and of potassium ions.

### Senescence

Previous studies have shown that both photosynthesis and Chl contents decline in senescent leaves. *GLK*s participate in the regulation of leaf senescence. It was identified that *ATAF1* regulates senescence through *GLK1* and *ORE1*. Rauf et al. (2013) verified that ORE1 and GLK proteins can specifically interact and form GLK1–ORE1 heteromers to repress expression of *GLK* target genes. Interestingly, micro164 is abundant in developing leaves and decreases during leaves senescence, and can repress *ORE1* expression (Kim et al., 2009). Determining whether micro164 controls *GLK*s will require further study. *GLK2* as the target gene of *PIF4* (*phytochrome-interacting factor 4*) was significantly repressed by *PIF4*; and *GLK1*, a target gene of *BZR1* and *PIF4*, was proposed to be regulated by them synergistically (Oh et al., 2012). Song et al. (2014) demonstrated that *PIF4* could repress *GLK2* expression and trigger senescence. Compared with wild type, *35S:GLK1* and *35S:GLK2* overexpressors showed delayed senescence, while *GLK1* and *GLK2* single mutants showed little change in their senescence behavior.

## *GLK*s and Phytohormones

Phytohormones play adjustment roles in plant growth and development. In several studies, phytohormones including auxin and brassinosteroids have been implicated in tomato fruit chloroplast development and correlated with increased *SlGLK* expression. In rice, *OsGLK1* regulates chloroplast development, which is also controlled by light and phytohormones, and *OsGLK1* plays roles in the late steps of Chl biosynthesis (Nakamura et al., 2009). Previous study identified that down-regulation of *DR12/SlARF4* in tomato may be related to the dark-green fruit phenotype (Jones et al., 2002). In *SlARF4*-silenced lines, the expression of *SlGLK1* and *SlGLK2* is elevated, indicating that *SlARF4* negatively regulates expression of *SlGLK1* and *SlGLK2* (Sagar et al., 2013; Nadakuduti et al., 2014). Several observations indicate that the relationship of *DDB1* and *GLK2* has an additive effect (Nguyen et al., 2014). The *bes1-D* seedlings are pale green and have a striking reduction in Chl. Subsequently, Yu et al. (2011) identified that *BES1* acts to repress expression of *GLK1* and *GLK2*, thereby adversely affecting chloroplast function. Whether *BES1* controls *GLK1* and *GLK2* expression through *PIL6* will be interesting to determine.

## Potential Targets of *GLK*s

Their primary target genes are light harvesting and Chl biosynthesis genes. *GLK*s directly bind to the promoters of target genes and act as transcriptional activators (Waters et al., 2009). *GLK*s interact with proline-rich regions of G-box-binding bZIP factors in *Arabidopsis* (Tamai et al., 2002). The G-box of light-regulated promoters is important for plastid retrograde signaling and *GLK*s are found to act downstream of the pathway. In addition, *K^+^ CHANNEL IN ARABIDOPSIS THALIANA1* (*KAT1*) may be a direct target of *GLK*s (Nagatoshi et al., 2016). Except their primary target genes are up-regulated in GLKs*_OX_*, so does GLK-non-targeted genes associated with chloroplast development.

## Ubiquitin-Conjugated Degradation of GLK Transcription Factors

Levels of proteins such as SlGLK2 are negatively regulated by ubiquitin–proteasome system (UPS)-mediated proteolysis in eukaryotes (Tomko and Hochstrasser, 2012; Tang et al., 2015). In tomato, Tang et al. (2015) demonstrated that *GLK2* associates with the CUL4–DDB1–DET1 E3 complex using co-immunoprecipitation and bimolecular fluorescence complementation. Two lysine residues (K11 and K253) of the GLK2 protein play an important role in the ubiquitin-mediated degradation of GLK2 (Tang et al., 2015).

## Conclusion and Perspectives

*GLK*s are members of the GARP family, conserved proteins that originated in bryophytes. Since the first isolation of *G2* in 1927, *GLK*s have been identified in many plants. Their best described role is chloroplast development, although they also participate in disease defense and senescence. Summarizing the history, conservation, function, and degradation of the GLK proteins, shows that little is known about the structure of GLKs or *GLK*s in perennial plants, and this area requires more study. Overall, *GLK*s, as part of chloroplast and nuclear genomes, control the development of chloroplasts, and determine the capacity for photosynthesis. Manipulating the expression patterns of *GLK*s may provide an opportunity to increase production and quality traits in many species.

## Author Contributions

MC, LL and DG designed the article. MC, MJ, BW, LiL, SL, XC wrote the manuscript and DG, LL revised the intellectual content of this manuscript. All authors read and approved the final manuscript.

## Conflict of Interest Statement

The authors declare that the research was conducted in the absence of any commercial or financial relationships that could be construed as a potential conflict of interest.
